# Development of a quantitative measurement on visual clutter in see through display

**DOI:** 10.3389/fnins.2023.1138225

**Published:** 2023-02-06

**Authors:** Qingfeng Liu, Yanyan Wang, Yu Bai, Mengsun Yu, Zhengtao Cao, Xinli Yu, Li Ding

**Affiliations:** ^1^School of Biological Science and Medical Engineering, Beihang University, Beijing, China; ^2^AirForce Medical Center, Fourth Military Medical University, Beijing, China

**Keywords:** visual clutter, eye track, cockpit, display, ergonomics

## Abstract

**Objective:**

With the wide use of transmission displays to improve operation performance, the display information highlights clutter because of the contradiction between the massive amount of information and limited display area. Our study aimed to develop a quantitative measurement for declutter design and appraisal.

**Methods:**

Using the ergonomics research system of characters and symbols in a see-through cockpit display, we set the simulated flight task interface at four pixel scale levels by enlarging all the display elements in a certain ratio. Flight task videos of 12 clutter degrees were recorded using each flight interface matched with three flight scene complexity levels. A total of 60 pilots completed the visual search tasks in the flight task video while the eye tracker was used to record the view path in real time. Visual search performance was analyzed to study the effect of various clutter factors and levels on pilots’ performance in visual search tasks, and acquire quantitative clutter measure parameters.

**Results:**

GLM univariate test revealed that there were significant differences among the fixation time in areas of interest (AOI), total Fixation point number, total fixation time at four pixel scale levels, and three flight scene complexity levels (*P* < 0.05). Visual search performance declined after the cutoff point, while the clutter degree increased. According to the visual search performance data, the recommend feature congestion upper pixel number limit in a 600*800 display was 18,576, and the pixel ratio was 3.87%.

**Conclusion:**

A quantitative measurement for declutter design and appraisal of cockpit displays was developed, which can be used to support see-through display design.

## 1. Introduction

Modern air combat involves system operation, during which, information regarding the war field state, the two-sided situation and the command order need to be interchangeable and allow comprehensive perception. Therefore, human aircraft interface design has become a key factor for operation performance. The narrow cockpit of a military fighter limits the display space. Innovative display technologies characterized by see-through displays such as the head-up display (HUD) (Betts, 1975; Doucette, 2012) and helmet-mounted display (HMD) (Newman and Greeley, 1997) have been applied to improve the operation performance. However, display information clutter has been highlighted because of the contradiction between the massive amount of information and the limited display area. Federal Aviation Administration (2014) pointed out that a cluttered display may result in an increased processing time for flight crew to obtain display information, so clutter should be minimized during display design. As the pilot must see through the HUD, special attention is needed to avoid display clutter that would otherwise unduly obscure the outside view. SAE International (1998) also stresses that a decluttered design is a necessary requirement for HUD.

Several clutter measures have been used in advanced cockpit displays. Subjective impressions of clutter may be collected with a multidimensional measure of clutter (Kaber et al., 2008) or overall perceived clutter rating (Doyon-Poulin et al., 2014). Rosenholtz et al. (2005) created an objective measure of clutter based on the feature congestion theory and image analysis technology. The image feature is calculated after being transformed to perceptual base International Commission on Illumination (CIE) lab color space and Gaussian pyramids. Kim et al. (2011) used this method to measure nine HUD configurations in a simulation landing flight of a civil aircraft. Consequently, the outside scene remained relatively stable and screenshot images of each configuration were extracted from videos recorded during the simulation flight and analyzed to calculate the clutter score. However, for fighter see-through displays, the outside view is successively changing during a maneuver task. Therefore the display element and flight scene, which are two clutter factors, could not be integrated into one image. Tullis (1983) proposed that active pixel numbers were an available method to measure the clutter of a black and white display. This method should be applied to see-through aviation displays since the border of characters and symbols are clear with no background.

In our study, we aimed to develop a quantitative measurement based on pixel numbers for declutter design and appraisal for see through displays in fighter cockpits.

## 2. Methods article types

### 2.1. Subjects

A total of 60 male pilots [mean (SD), age 22.14 ± 9.24 y, flight hours 1,250.68 ± 1,522.84 h] participated the experiment. All subjects were medically qualified. The study was approved by the Logistics Department of the Civilian Ethics Committee of Beihang University. All subjects who participated in the experiment were provided with and signed an informed consent form. All relevant ethical safeguards were met with regard to subject protection.

### 2.2. Equipment and test setting

#### 2.2.1. Experiment flight task design

An ergonomics research system of display characters and symbols in the military cockpit was developed based on an analysis of display factors, layout and arrangement of the interface in modern military cockpits using Microsoft Visual Studio, 2013 edit. Display elements, the vector data set and the typical flight visual scene database were edited. We designed and edit the display elements including shape, size, location, color, salience etc., and recorded a flight task video with a dynamic flight scene using ergonomics evaluation and research. The total pixels of each and all elements were calculated by the program.

**FIGURE 1:**
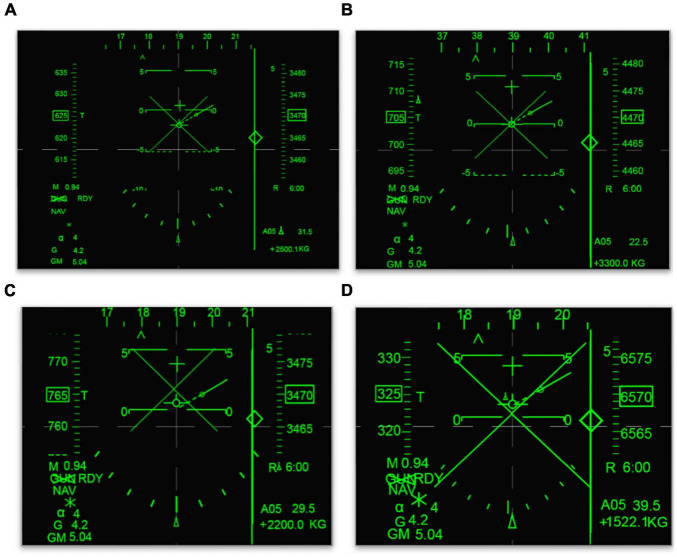
Four clutter level displays. **(A)** Clutter level 1. **(B)** Clutter level 2. **(C)** Clutter level 3. **(D)** Clutter level 4.

**FIGURE 2:**
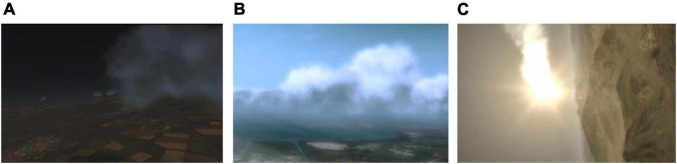
Dynamic flight scene videos of night, day, and complex environment. **(A)** Night scene. **(B)** Day scene. **(C)** Complex environment scene.

**FIGURE 3:**
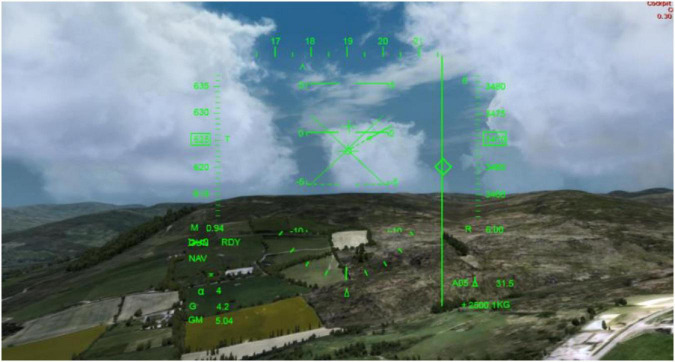
Screenshot of dynamic flight scene videos.

Using an ergonomics research system, we set simulation flight task images at four clutter levels by enlarging all of the display elements in a certain ratio (Figure 1). A target symbol 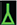 was inserted in each display image for the search task. Dynamic flight scene videos of three complexity levels (night, day and a complex environment) were constructed (Figure 2). Flight task videos of 12 clutter degrees were recorded using each flight interface matched with three complexity levels flight scenes (Figure 3). The video format was Windows Media Video (WMV) and the duration was 45 s. A 4 × 3 experiment design was used.

#### 2.2.2. Eye tracker

A Tobii Pro X2-30 eye tracker was used in the experiment. This is a small, full-feature eye tracking system that can be mounted below the PC monitor. The fixation angle could be up to 36°.

#### 2.2.3. Procedure

The pilots signed a statement of informed consent, which outlined the purpose of the experiment and informed the subjects of their rights when they were recruited. The pilots were seated 70 cm in front of a computer monitor with a Tobii X2-30 eye tracker mounted below the screen. They were given instructions and completed the eye tracker calibration. Then, they completed three practice trials to ensure the correct operation of the search task. Pilots then pressed the enter key on the keyboard to start the search task. They were asked to find the given target as soon as possible and press the enter key when they found it. Then, the next trial would begin. Each pilots completed 48 trials. The eye tracker recorded the eye movement behavior and fixation time in areas of interest (AOI), and total fixation point numbers, total fixation time, and saccade length were analyzed. The experiment was carried out in daylight with normal laboratory illumination.

### 2.3. Statistical analysis

The Statistical Product and Service Solutions (SPSS) 19.0 statistical software package was used to analyze the data. All the test data were expressed as M or M ± SD (s). The general linear model (GLM) univariate test, χ^2^ test, and Pearson correlation were used for analysis; *P* < 0.05 was set as the threshold value of significant difference in the statistical analysis.

## 3. Results

### 3.1. Descriptive analysis

As eye tracking data including fixation time in AOI, total fixation point numbers and total fixation time did not conform with the normal distribution, log transformation was carried out to normalize the data. The data of the mean saccade length were near the normal distribution.

### 3.2. Clutter effects of varied clutter levels and flight scene complexity levels

**FIGURE 4:**
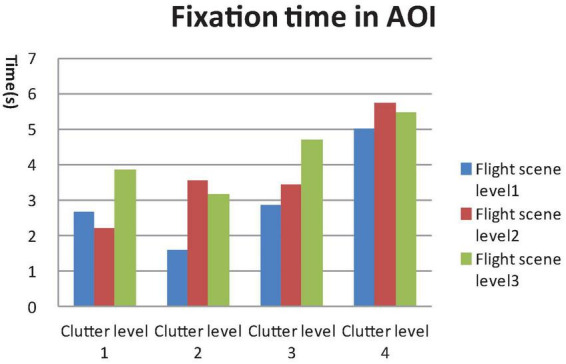
General linear model (GLM) Univariate test of fixation time in areas of interest (AOI).

**FIGURE 5:**
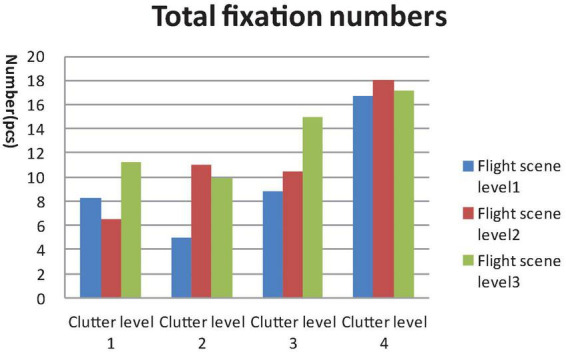
General linear model (GLM) Univariate test of total fixation numbers.

**FIGURE 6:**
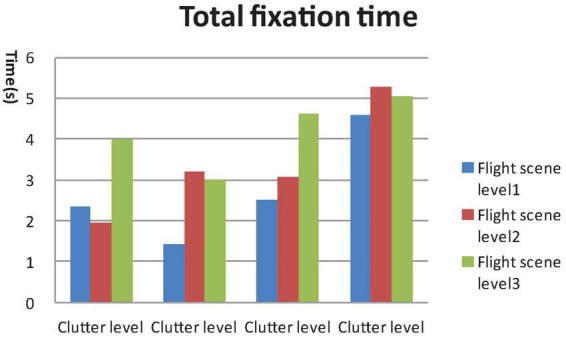
General linear model (GLM) Univariate test of total fixation time.

**FIGURE 7:**
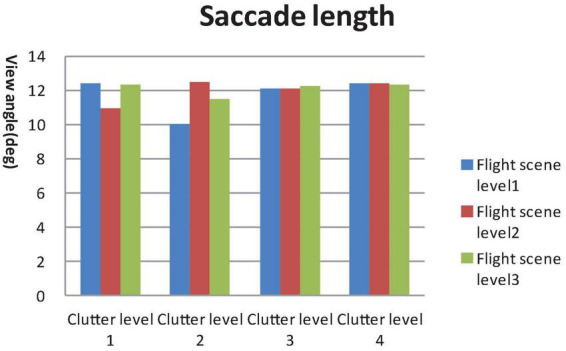
General linear model (GLM) Univariate test of saccade length.

A 4 × 3 GLM univariate test revealed that the fixation time in AOI, total fixation numbers and total fixation time were significantly different among the four clutter levels [F_Fixation time in AOI_ (4, 812) = 47.907, F_Toatal Fixation point number_ (4, 812) = 46.714, F_Total Fixation time_ (4, 812) = 49.556, *P* < 0.01) and three flight scene complexities (F_Fixation time in AOI_ (4, 812) = 21.402, F_Toatal Fixation number_ (4, 812) = 16.878, F_Total Fixation time_ (4, 812) = 32.743, *P* < 0.01]. The mean saccade lengths were significantly different among the four clutter levels [F_Mean saccade length_ (4, 812) = 3.179, *P* < 0.05], but not the three flight scene complexities [F_Mean saccade length_ (4, 812) = 0.676, *P* > 0.05], as shown in Figures 4–7.

Results of the *post hoc* least significant difference (LSD)-t on clutter levels indicated that there were no significant differences between level 1 and level 2 in terms of all eye tracking data (*P* > 0.05). The fixation time in AOI, total fixation number and total fixation time data for levels 3 and 4 were higher than those of levels 1 and 2. The saccade length data of level 4 were higher than those of level 1 and level 2 (*P* < 0.05). The *post hoc* LSD-t on flight scene complexity levels indicated that the fixation time in AOI, total fixation point number and total Fixation time data of level 2 were higher than level 1, and those of level 3 were higher than level 2 (*P* < 0.01). Therefore, the visual search performance declined after the cutoff point while the clutter degree (pixel factor and flight scene factor) increased.

### 3.3. Analysis of delayed reaction

In the experiment, the duration of each task video was 45 s, which was long enough for the target search. In a real flight task, the available search time maybe transient because of the rapid speed of the aircraft. Therefore, we set 10 s as the cutoff value which meant that tasks with a search time higher than 10 s were invalid or represented a delayed reaction. The delayed reaction rate is important for search performance just as the error rate is important for a reaction time task. The χ^2^ test showed that delayed reaction numbers increased with the clutter degree (χ^2^ = 53.390, *P* = 0.000), as shown in Table 1.

**TABLE 1:** Delayed reaction of varied clutter degrees.

	Clutter level 1	Clutter level 2	Clutter level 3	Clutter level 4
>10 s	2	8	14	40
<10 s	196	203	189	161

### 3.4. Quantitative clutter measure parameters calculation

According to visual search performance data, method of linear interpolation was used to calculate the quantitative parameters. Table 2 presents pixel numbers and pixel ratio of each clutter levels. Table 3 presents estimated marginal mean of Fixation time in AOI which is equal to searching time and will be used to calculate the clutter measure parameters.

**TABLE 2:** Estimated marginal mean of Fixation time in (AOI) (s).

Clutter levels	Mean reaction time (RT, s)	SE	CI 95% Upper	CI 95% lower
Level 1	2.831	1.049	2.578	3.109
Level 2	2.610	1.047	2.387	2.854
Level 3	3.585	1.049	3.266	3.934
Level 4	5.408	1.049	4.924	5.940

**TABLE 3:** Pixel numbers and pixel ratio of each clutter levels.

Clutter levels	Pixel number (PN, px)	Pixel ratio (PR, %)
Level 1	10,254	2.14
Level 2	15,631	3.26
Level 3	21,545	4.49
Level 4	28,353	5.91

According to the GLM univariate test results, the search performance decreased after clutter level 2, since the eye tracking data showed no significant difference between level 1 and level 2, but did show a significant difference between level 2 and level 3. The clutter cutoff point should therefore be between level 2 and level 3.

According to method of linear interpolation:

The clutter of pixel numbers (CPN) cutoff point formula is:

CIR = AVE (PN_2_ + PN_3_)

∵ PN_2_ = 15,631, PN_3_ = 21,545

∴ CPR = AVE (15,631 + 21,545) = 18,756

The clutter of pixel ratio (CPR) cutoff point formula is:

CIR = AVE (PR_2_ + PR_3_)

∵ PR_2_ = 3.26, PR_3_ = 4.49

∴ CPR = AVE (3.26 + 4.49) = 3.87%

The clutter of reaction time (CRT) cutoff point formula is:

CRTP = AVE (RT_2_ + RT_3_)

∵RT_2_ = 2.610, 95% CI = 2.387, 2.854

RT_3_ = 3.585, 95% CI = 3.266, 3.934

∴CRTP = AVE (2.610 + 3.585) = 3.097 s, 95% CI = 2.827, 3.394

The results revealed that the recommended feature congestion upper pixel number limit for a 600*800 display was 18,576 px, and the pixel ratio was 3.87%. When using a search task to evaluate the clutter of a see-through display, the search time should not be over 3.097 s, 95% CI = 2.827, 3.394.

## 4. Discussion

Clutter is a key concern in the design of aviation displays because it is not only a perceived crowding sense, but also a potential negative factor on flight performance, especially in fighter see-through displays whose scale is limited by the cockpit space. Though it is widely accepted that human performance is particularly sensitive to visual clutter in searching tasks, results of studies on aviation display visual clutter are somewhat contradictory. Ververs and Wickens (1998) found that a cluttered display could cause a little longer change detection time, but have no effect on flight performance. Doyon-Poulin et al. (2014) found that middle clutter display can led to better simulation flight performance, as measured by localizer deviation, but this effect was not found in other flight indices. Kim et al. (2011) also suggested that middle clutter HUD configurations were better for a pilot’s landing performance, and that cognitive complexity and a lack of information for high and low clutter displays may cause higher workload and less stable operation, respectively. Rarely, studies have focused on fighter see-through displays, and middle clutter is still a concept with less quantitative criterion. Subjective rating is the most common clutter evaluation method for aviation displays since both the visual density and task-relevant dimensions of clutter are considered. The disadvantage is that subjective evaluation can only be carried out in a later design process, when the displays have been provided with their main functions.

Our study aimed to develop a quantitative measurement for declutter design and appraisal that can be used in the early design stage of see-through displays in fighter cockpits. Pixel numbers and ratio were selected as the quantitative measurements for see-through display clutter for three reasons.

Firstly, active pixel numbers is a valid visual clutter measurement on information density. Grahame et al. (2004) measured webpage clutter in pixels as a percentage of the total page space that was occupied by meaningful elements. This method should be applied to see-through aviation displays since the borders of characters and symbols are clear with no colorful background. Nickerson (1994) also suggested that the ratio of the space that is occupied by meaningful units to that of background can evaluate information clutter. Frank and Timpf (1994) even used ink dosage to measure the information density on a black and white map.

Secondly, this simple method is sufficient for fighter see-through display clutter measurement. There are several image analysis methods and software for clutter measurement, for example, Insight (Arents et al., 2010). Display image properties such as color, luminosity, and orientation can be analyzed by an image processing program. Rosenholtz et al. (2005, 2011) has proposed a feature congestion theory and a measure of display clutter and validated such tools during the aviation display design process. Finally, the outside flight scene is difficult to measure using image analysis methods since the fighter moves successively in multi-degrees and the outside scene changes rapidly during a maneuver. Therefore, the flight scene may have an effect on see-through display clutter and it cannot be measured by image analysis.

We develop an ergonomics research system of display characters and symbols in a military cockpit, where we designed and constructed simulation flight task images of four pixel levels by enlarging all the display elements in a certain ratio, and dynamic flight scene videos of three complexity levels (night, day, and a complex environment). An eye tracker was used to record visual search behaviors. The experiment using pilots showed that the visual search performance declined after the cutoff point, while the clutter degree (pixel number factor) increased. The flight scene complexity had a negative effect on visual clutter. Delayed reaction numbers increased gradually as clutter degree grew. The ratio even arrived at 20% at clutter level 4 in the experiment, which is totally unacceptable for military flights. Higher active pixel numbers or ratio should be avoided in see-through display design.

According to the visual search performance data, the method of linear interpolation was used to calculate the quantitative parameters. Feature congestion and performance indices were calculated. Feature congestion measured by pixel number reflected the information density, which can guide design directive. The performance index acquired from the eye tracker can be used in ergonomics evaluation by pilots. The recommended upper limit of the pixel number in a 600*800 display is 18,576 px, and the pixel ratio is 3.87%. When using a search task to evaluate the clutter of a see-through display, the search time should not be over 3.097 s, 95% CI = 2.827, 3.394.

In our study, a quantitative measurement based on pixel numbers was developed and validated for declutter design and appraisal of see-through cockpit displays. The method could be used to evaluate the clutter level of interfaces for ergonomic improvement.

## Data availability statement

The original contributions presented in this study are included in the article/supplementary material, further inquiries can be directed to the corresponding author.

## Ethics statement

The studies involving human participants were reviewed and approved by the Logistics Department of the Civilian Ethics Committee of Beihang University. The patients/participants provided their written informed consent to participate in this study.

## Author contributions

QL: conceptualization, methodology, experiment, formal analysis, and writing—original draft. YW: data curation, methodology, experiment, and formal analysis. YB: experiment and data curation. MY: resources and supervision. ZC: experiment and validation. XY: visualization and writing—review editing. LD: conceptualization, funding acquisition, resources, supervision, and writing—review editing. All authors contributed to the article and approved the submitted version.
